# Sensitive mass spectrometric assay for determination of 15-deoxy-Δ^12,14^-prostaglandin J_2_ and its application in human plasma samples of patients with diabetes

**DOI:** 10.1007/s00216-017-0748-1

**Published:** 2017-11-16

**Authors:** Jakob Morgenstern, Thomas Fleming, Ivelina Kadiyska, Sebastian Brings, Jan Benedikt Groener, Peter Nawroth, Markus Hecker, Maik Brune

**Affiliations:** 1 Department of Internal Medicine I and Clinical Chemistry, University Hospital Heidelberg, Im Neuenheimer Feld 410, 69120 Heidelberg, Germany; 2 German Center for Diabetes Research (DZD), Ingolstädter Landstraße 1, 85764 Neuherberg, Germany; 3 Institute of Physiology and Pathophysiology, Division of Cardiovascular Physiology, University of Heidelberg, Im Neuenheimer Feld 326, 69120 Heidelberg, Germany; 4 Department of Nuclear Medicine, University Hospital Heidelberg, Im Neuenheimer Feld 400, 69120 Heidelberg, Germany

**Keywords:** Mass spectrometric assay, 15-Deoxy-Δ^12,14^-prostaglandin J_2_, Human plasma samples

## Abstract

The determination of individual prostaglandins (PG) in humans is mainly performed in urine samples. The quantification of PGs in human plasma could improve the understanding of particular PG species under various physiological and pathological conditions. 15-Deoxy-Δ^12,14^-prostaglandin J_2_ (15d-PGJ_2_) is a dehydrated downstream product of PGD_2_ and is of high interest due to its recently discovered anti-inflammatory effects. Increasing availability of highly sensitive mass spectrometry allows the quantification of low abundant biomarkers like 15d-PGJ_2_ in human plasma samples. Herein, a sensitive LC-MS/MS method for the determination of 15d-PGJ_2_ was established. The method was validated according to the guidance of the American Food and Drug Administration and tested in plasma samples from patients with poorly controlled diabetes, considered to be a pro-inflammatory condition. Extraction of 15d-PGJ_2_ was achieved with an easy-to-use liquid-liquid extraction by ethyl acetate following a methanol precipitation. The lower limit of quantification was 2.5 pg mL^−1^ and linearity (*R* ^2^ = 0.998) was guaranteed between 2.5 and 500 pg mL^−1^ for 15d-PGJ_2_. Selectivity was assured by the use of two individual mass transitions (qualifier and quantifier). Precision and accuracy were validated in an inter- and intraday assay with a coefficient of variation below 11.8% (intraday) and 14.7% (interday). In diabetic patients with an HbA_1C_ > 9%, increased plasma concentrations of 15d-PGJ_2_ compared to control plasma were measured. 15d-PGJ_2_ correlated negatively with the inflammation marker C-reactive protein. The developed LC-MS/MS method represents a new possibility to quantify 15d-PGJ_2_ with high specificity in human plasma samples. This may contribute to a better understanding of the potential anti-inflammatory effects of 15d-PGJ_2_ in severe long-term pro-inflammatory disorders like diabetes, cancer, or cardiovascular disease.

**Electronic supplementary material:**

The online version of this article (10.1007/s00216-017-0748-1) contains supplementary material, which is available to authorized users.

## Introduction

Prostaglandins (PGs) represent an important class of bioactive lipid metabolites which have been extensively investigated in the last decades. Considerable attention has been paid to the subtypes PGE_2_ and PGI_2_ which are involved in numerous pathways promoting different states of inflammation or regulating coagulation [1]. Imbalances in their synthesis are associated with abnormal immune function but also with deregulated dilation and constriction of vascular smooth muscle cells as well as disrupted platelet aggregation [1–3]. It has been shown that 15-deoxy-Δ (12,14)-prostaglandin J2 (15d-PGJ_2_), a downstream product of PGD_2_, is potentially involved in immune responses. This metabolite has been identified as the endogenous ligand of the nuclear receptor PPARγ, which is implicated in the normalization of inflammatory processes. Given this property, 15d-PGJ_2_ could be important in the resolution of acute and long-term inflammation and therefore affect the pathogenesis of inflammatory disorders [4–6].

Most of the PGs have a relatively short half-life since they are rapidly metabolized and excreted [7, 8]. This is one of the main reasons why many clinical investigations of PGs were based upon the analysis of urinary degradation products [9]. Circulating PGs are degraded rapidly and metabolites (e.g., t-PGDM, t-PGEM, t-PGFM) are excreted via the kidney [10]. Studies regarding PGE_2_ and PGF_1α_ have also shown that urinary prostaglandins are a reflection of renal prostaglandin synthesis and do not reflect circulating levels of these PGs [11, 12]. Another handicap of urine quantification is the need for normalization to urinary creatinine levels as to account for differences in diuresis, despite creatinine levels being highly dependent on diet, gender, age, muscle mass, and physical activity. In addition to the determination of urinary PG levels, quantification of PGs in plasma would be a rapid and useful way to validate the PG metabolome. The determination of PGs in plasma also offers the possibility for clinicians to observe and adjust anti-inflammatory treatment regimes. Currently, studies of PGs in human blood samples were mainly done by enzyme- or radioimmunoassays. Low sensitivity and limited specificity as well as moderately high costs due to substantial amounts of antibodies are obvious disadvantages for routine clinical practice [reviewed in 13]. A higher sensitivity can be obtained with gas chromatography coupled to mass spectrometry, but complex derivatization steps are needed, which is a limiting factor when processing large amounts of samples [14, 15]. State of the art techniques such as LC-MS/MS achieve high sensitivity and selectivity and can quantify low abundant biomarkers more accurately. Such technology offers the opportunity for a direct snapshot of PGs circulating in the human blood at pico- and nanomolar levels [16].

The aim of this study was to establish a robust and simple LC-MS/MS method for the quantification of 15d-PGJ_2_ in human plasma samples. This includes criteria such as high specificity with two mass transitions (qualifier/quantifier), a short runtime (10 min), satisfying accuracy and an inexpensive liquid-liquid extraction (LLE) with recovery yields above 80%. In order to study the role of 15d-PGJ_2_ under a pro-inflammatory condition such as diabetes, the developed method was applied to a cohort of patients with poorly controlled diabetes (HbA_1c_ > 9%).

## Materials and methods

### Chemicals and reagents

Acetonitrile, ammonium acetate, ethyl acetate, methanol, and water were high purity grade and purchased from Sigma-Aldrich (Steinheim, Germany). Formic acid was purchased from Biosolve (Valkenswaard, Netherlands). 15-Deoxy-Δ^12,14^-prostaglandin J_2_ (15d-PGJ_2_) and 15-deoxy-Δ^12,14^-prostaglandin J_2_-d4 (d4-15d-PGJ_2_) were purchased as LC-MS standard (purity > 98%) from Cayman Chemical (local distributor: Biomol, Hamburg, Germany). Artificial plasma with a standardized protein composition of human plasma (Biseko®) was purchased from Biotest (Dreieich, Germany) and was used as a matrix for method development and validation. A liter Biseko© solution contains 50 g total protein, including albumin (31 g), IgG (7.1 g), IgA (1.55 g), IgM (0.48 g), sodium ions (3.56 g), potassium ions (0.16 g), calcium ions (0.08 g), magnesium ions (0.02 g), chloride ions (3.65 g), and water for injections.

### Preparation of calibration standards

All standard solutions were evaporated to dryness under a gentle nitrogen flow and stored in methanol at − 80 °C at concentrations ranging from 100 ng mL^−1^ to 100 μg mL^−1^. Working solutions were prepared in methanol and kept at − 80 °C. A small aliquot (0.5 mL) was stored in dimethyl sulfoxide (DMSO), water, and ethanol to compare the long-term stability of PGs in these matrices at − 80 °C. Calibration standards of 15d-PGJ_2_ were 2.5, 5, 10, 50, 100, and 500 pg mL^−1^ + 0.1 ng d4-15d-PGJ_2_ for each calibrator.

### Sample collection and extraction

EDTA plasma samples from 20 healthy controls and 25 type 2 diabetic patients were obtained. All participants were in a fasting state and for diabetic patients the inclusion criterion was an HbA_1C_ value above 9%. The study was approved by the ethics committee of Heidelberg University Hospital. All patient material and data was acquired with formal written informed consent and in agreement with the guidelines of the ethics committee. Packed red blood cells from five healthy controls were isolated via gradient centrifugation (Ficoll-Paque™, GE Lifescience, Freiburg) in order to study the hemolysis effect. Hemoglobin concentrations were determined using a commercially available Drabkin-Assay (Sigma, Steinheim, Germany).

Plasma samples were immediately aliquoted following centrifugation (5000×*g*, 5 min, 4 °C) of the whole blood and freshly frozen at − 80 °C. For LLE, 50 μL of internal standard (IS) (0.1 ng d4-15d-PGJ_2_) was added to 500 μL of human/artificial plasma, which was then acidified with 5 μL of formic acid to obtain pH 2–3. Afterwards, 200 μL of methanol was added to achieve plasma protein precipitation. After centrifugation (10 min at 14,000×*g*; 4 °C), 500 μL of ethyl acetate was added to the supernatant and vigorously mixed. Aqueous and organic phases were separated by centrifugation (10 min at 14,000×*g*; 4 °C) and the procedure was repeated twice. The organic ethyl acetate phases were combined and evaporated using a vacuum concentrator (Savant SpeedVac™ SC100) at room temperature. The residue was resuspended in 100 μL of a mixture of acetonitrile-water (1:1) with the addition of 0.1% ammonium acetate and after a short spin down (1 min at 14,000×*g*; 4 °C) 80 μL were transferred into an HPLC vial for further analysis.

### Chromatography

All analyses were performed on a Waters® Acquity UPLC class I system (Waters, Eschborn, Germany) equipped with a binary solvent delivery system with an online degasser and a column manager containing a column oven connected to an UPLC autosampler. PGs were separated by reverse-phase LC on a Waters® Acquity BEH C18 column (1.7 μM, 2.1 × 50 mm) at a flow rate of 0.3 mL min^−1^ and a column temperature of 40 °C. During analyses, all samples were stored in the autosampler at a temperature of 4 °C and the injection volume for each sample varied between 1 and 10 μL. Solvent A consisted of 0.1% ammonium acetate in water and solvent B was 0.1% ammonium acetate in a mixture of acetonitrile/water (95:5). For each run, a gradient elution was performed and no pre-equilibration was needed: 0 ➔ 2 min, 75 ➔ 70% solvent A; 2 ➔ 2.5 min, 70 ➔ 5% solvent A; 2.5 ➔ 8 min, 5 ➔ 70% solvent A; 8 ➔ 10 min, 70 ➔ 75%. The column eluent was directed into the MS and analyses were performed using *MassLynx XS* software.

### Mass spectrometry

The detection of 15d-PGJ_2_ was carried out on a XEVO TQ-S tandem quadrupole mass spectrometer (Waters®) equipped with an electrospray ionization source (ESI) operated in negative ion mode. Analyte detection was performed using multiple reaction monitoring (MRM). Source parameters were set as follows: capillary voltage 3.8 kV, desolvation temperature 300 °C, desolvation gas flow 850 L/h, source temperature 150 °C, cone gas flow 250 L/h, collision gas flow 0.15 mL min^−1^, and nebulizer gas flow 5 bar. Cone and collision voltage were optimized for 15d-PGJ_2_ and d4-15d-PGJ_2_ separately and are summarized in Table 3. Acquisition and quantification was completed with *MassLynx* 4.1 and *TargetLynx* 2.7.

### Validation procedure

The method was validated according to guidance of the Food and Drug Administration (FDA) for a partial validation for bioanalytical methods [17]. Briefly, the method was validated for selectivity, matrix effects, linearity, lower limit of detection (LLOD) and quantification (LLOQ), recovery, stability, precision, and accuracy (intra-/interday). Linearity was evaluated based on spiked plasma samples (artificial plasma) with six different concentrations. Additionally, blank samples (without analytes) and zero samples (only IS) were measured for each calibration curve to ensure reliability. A six-point calibration was performed using linear regression by adding increasing amounts of each standard and constant amounts of the IS. For all concentration calculations, the area ratio of compound/IS was plotted against nominal calibrator concentration. For the determination of recovery, LLOD, LLOQ, precision, and accuracy artificial human plasma (500 μL) employing the described LLE method were used. Recovery, precision, and accuracy were validated in an intraday assay using three different concentrations of the calibration range (low, mid, high) and measured in quadruplicates. The extraction recovery at low, medium, and high levels of QC samples was obtained using the following equation: $\mathrm{Recovery}=100\times \frac{\mathrm{Measured}\kern0.17em \mathrm{concentration}}{\mathrm{Nominal}\kern0.17em \mathrm{concentration}}$. For the interday variability, artificial human plasma was spiked with three different concentrations (low, mid, high) within the calibration range and examined on three consecutive days measured in quadruplicates for each concentration. LLOD and LLOQ were determined by definition of a signal-to-noise ratio (*S*/*N*) of 6 (LLOQ) and 3 (LLOD). Stability was validated in human whole blood (pre-processed) and in assay buffer (post-processed) at various temperatures and for different durations. Matrix effects were defined as a suppression or increase of signal intensity for the chosen MRMs (matrix effects while ionization) or as an increase or decrease in recovery of the IS (matrix effect while extraction). In this context the six-point-calibration in a blank matrix (water) was compared with the same calibration carried out in a biological matrix (artificial plasma). For studying hemolysis effect, spiked artificial plasma samples (150 pg mL^−1^ of 15d-PGJ_2_) were additionally spiked with lysed erythrocytes at three different concentrations of hemoglobin (10, 50, 500 mg dL^−1^).

### Determination of C-reactive protein

Plasma levels of C-reactive protein (CRP) in all patients were analyzed with an immunoturbidimetric assay on an ADVIA 2400 chemistry analyzer (Siemens) according to the standard operating protocol in the central laboratory of Heidelberg University Hospital.

### Statistical analysis

All data are expressed as mean values ± standard error (SE) and were analyzed for significance using unpaired *t* test with Welch’s correction. Spearman correlation was used to study the association of CRP and quantified 15d-PGJ_2_ in type 2 diabetic patients.

## Results and discussion

### Fragmentation and mass transitions

**Fig. 1:**
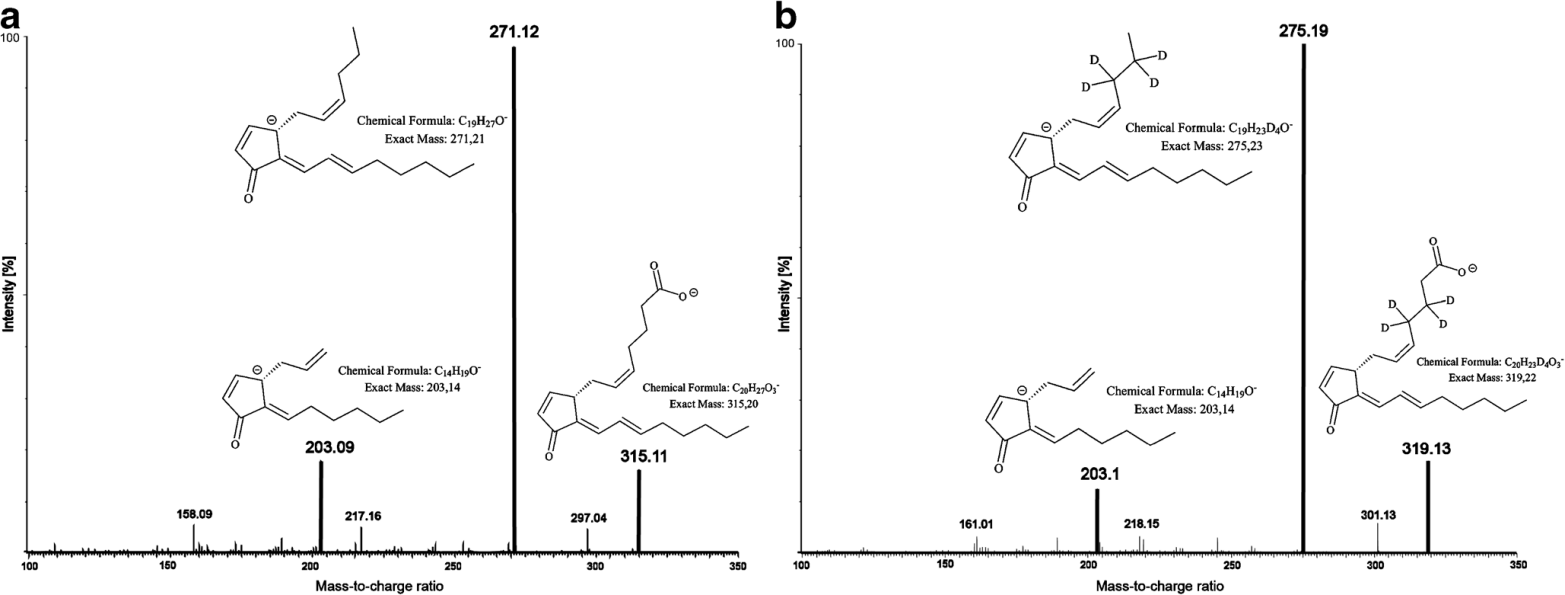
Total ion spectra and proposed fragmentation patterns for 15d-PGJ_2_ and its internal standard d4-15d-PGJ_2_. **A** Direct injection of 15d-PGJ_2_ (0.1 pg; COV 35 V; CE 15 V). **B** Direct injection of d4-15d-PGJ_2_ (0.1 pg; COV 33 V; CE 14 V)

Acquisition of 15d-PGJ_2_ was achieved in negative ESI mode, whereas positive ESI mode resulted in minor product ion intensity. Product ions were in accordance to previous published reports and are listed in Table 1 [18]. For 15d-PGJ_2_, daughter fragments of 271.1 *m*/*z* due to the loss of 1 molecule of carbon dioxide and *m*/*z* 203.1 (loss of five carbons) were detected (Fig. 1a). Higher signal intensity for the daughter fragments was achieved by reducing the cone/desolvation gas flow from 400 to 250 L h^−1^ or 1000 to 850 L h^−1^, respectively. Reduction of the nebulizer gas flow pressure (7 to 5 bar) and total injection volume from 5 to 2 μL was associated with an increase in parent ion signal intensity. Following optimization of the device specific parameters (Table 1), several injections of spiked biological matrix samples (artificial plasma) showed that fragmentation patterns for qualifier and quantifier of 15d-PGJ_2_ were reproducible and stable over a long period of time (*n* = 200).

**Table 1:** Retention times (*R*_t_), mass transitions (MRM), cone voltages (COV), and collision energies (CE) for 15d-PGJ_2_ and IS

Analyte	*R*_t_ [min]	MRM quantifier (*m*/*z*)	MRM qualifier (*m*/*z*)	COV [V]	CE [V]
15d-PGJ_2_	3.90	315.1 > 271.1	315.1 > 203.1	35	15
d4-15d-PGJ_2_	3.88	319.1 > 275.2	319.1 > 203.1	33	14

### Chromatography

**Fig. 2:**
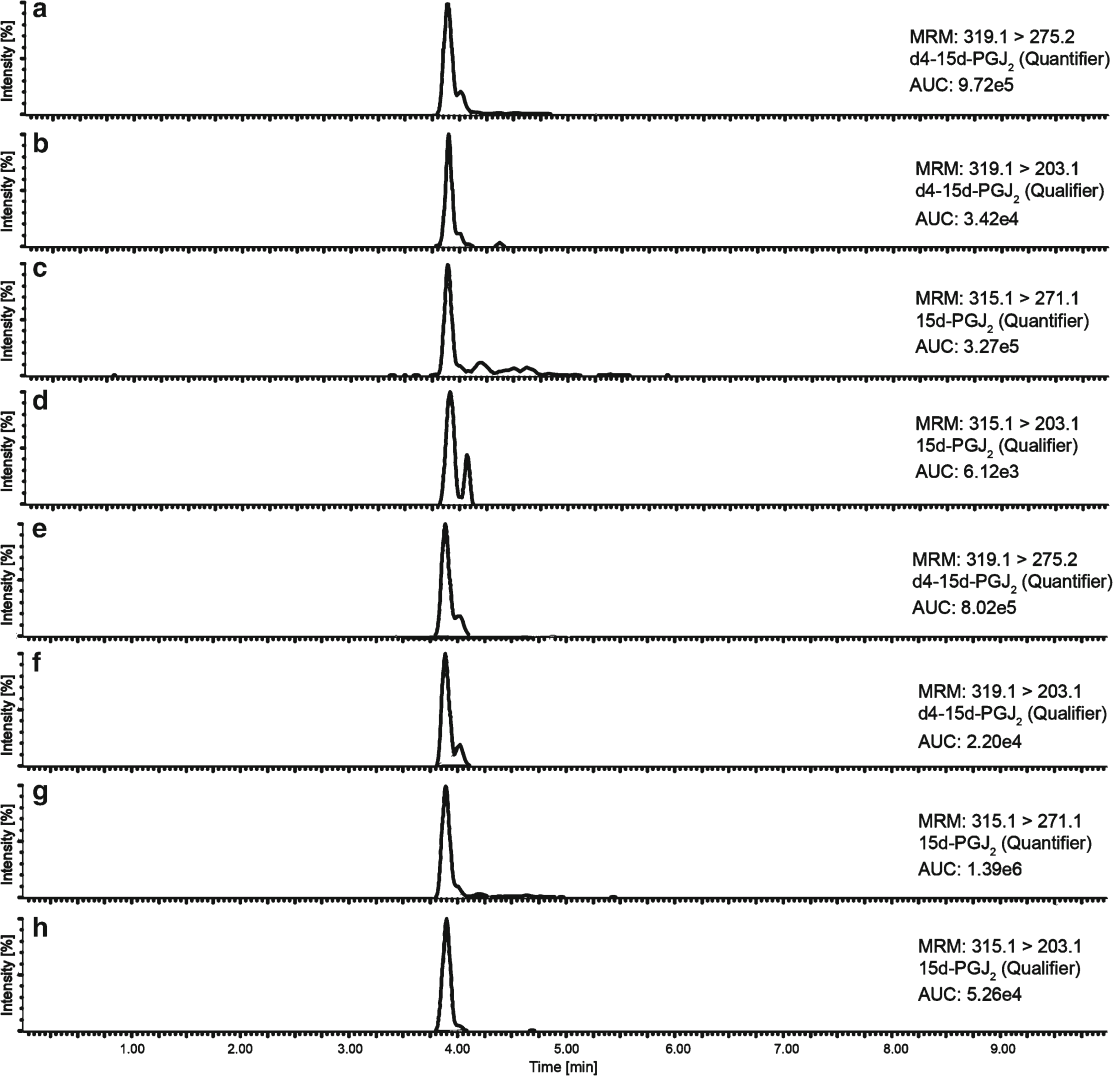
Extracted MRM chromatograms (quantifier/qualifier) of 15d-PGJ_2_ and its corresponding internal standard d4-15d-PGJ_2_ of a control sample (no. 3) with 17.50 pg mL^−1^ 15d-PGJ_2_ plasma concentration (**A**–**D**) or a type 2 diabetic patient (no. 8) with 136.82 pg mL^−1^ 15d-PGJ_2_ plasma concentration (**E**–**H**)

Previous studies have documented that reverse-phase columns are the most suitable for the separation of eicosanoids and PGs [18, 19]. Based on these data, a method was established with different BEH-C18-RP columns of the AQUITY® series from Waters®. Using a short column with the lowest particle size available (50 × 2.1 mm; 1.7 μm), it was possible to reduce the total runtime to 10 min. Usage of longer columns (100 or 150 mm), higher particle size (2.5 μm), as well as the combination of a pre-column (VanGuard™; 5 × 2.1 mm; 1.7 μm) produced closely eluting second peaks and intensified matrix interferences (see Electronic Supplementary Material (ESM) Fig. S1). The negative charge of precursor ions was enhanced during ionization by the addition of ammonium acetate instead of ammonium hydroxide to solvents A and B (~ 20% higher intensity for quantifier and qualifier product ions) as it has been done in previous approaches [16]. Different solvent compositions were tested in order to achieve a clean, sharp, and well-separated chromatogram. The short runtime and gradient elution with the usage of acetonitrile as solvent B significantly improved the chromatogram regarding intensity, peak shape, and reproducibility (Fig. 2a–h). Nevertheless, we observed the occurrence of increased LC system pressure and non-specific peaks during blank injections (maximum 10% intensity of lowest calibration standard) after the measurement of ~ 100 plasma samples. To decrease these phenomena, a 60-min blank run with H_2_O/ACN (1:1) with no additives was used after 50 injections to decrease the described problems. This procedure reduced high column pressure and the occurrence of ghost peaks (ESM Fig. S2).

### Analytical specificity

Proof of purity of chemical standards was provided by the supplier company. Two MRMs for 15d-PGJ_2_ and the respective IS were established (Table 1). Daughter ions yielding the highest intensity were selected as quantifier. For the quantification of 15d-PGJ_2_, the ratio of quantifier to qualifier was used to ensure reliable data, as it has been done before [19]. In the absence of endogenous 15d-PGJ_2_ level (artificial plasma blanks using Biseko®), no co-eluting compounds were found which interfered with the detection. Biseko® is a virus-inactivated human plasma that contains the entire spectrum of serum proteins in a standardized, active form. It is prepared from plasma pools of at least 1000 individual donations. Pooling of such large numbers of donations balances out individual differences in protein concentrations; however, the possibility that any medications could interfere with our assay could not be excluded [20].

### Linearity and determination limits

For the six-point calibration, the ratio between compound and IS was used for quantification. The obtained calibration coefficients (*R* ^2^) were > 0.998. Within the context of LLOD and LLOQ, acceptable values (Table 2) were achieved in comparison to other published methods in the field [13, 14, 21].

**Table 2:** Parameters of quantification

Analyte	Calibration range [pg mL^−1^]	Calibration coefficient [*R* ^2^]	LLOD [pg mL^−1^]	LLOQ [pg mL^−1^]
15d-PGJ_2_	2.5–500	0.9979	0.5 (*S*/*N* = 3.4)	2.5 (*S*/*N* = 10.9)

Displayed is the calibration range and coefficient (*R* ^2^), lower limit of detection (LLOD), and quantification (LLOQ) with appropriate signal-to-noise ratio (*S*/*N*) for each analyte

### Recovery, precision, and accuracy

Recovery for 15d-PGJ_2_ was between 92.1 ± 8.2% (Table 3). The precipitation of plasma proteins with methanol and acidification with formic acid prior to LLE displayed the highest yields in recovery. Stronger precipitation agents such as acetone (recovery ~ 59 ± 13.9%) or trichloroacetic acid (recovery ~ 75 ± 22.4%) were not able to improve these yields. The acidification with hydrochloric acid or other acidification reagents (acetic acid, trifluoracetic acid) was associated with the occurrence of anionized adducts (chloro-, fluoro-) in the mass spectrum and therefore with lower recoveries. Precision of replicate analyses was evaluated for three concentrations within the calibration curve. The coefficient of variation (CV) for intraday measurements was 8.1–11.8%, while for interday measurements, the CV was 6.1–14.7%. Accuracy was between 91 and 115%. Recovery and parameters of imprecision are summarized in Table 3. Hemolysis, even at very high concentrations of 500 mg dL^−1^ hemoglobin, had no effect on the recovery. However, only at this high concentration of hemoglobin we found that there was a significant increase in signal for 15d-PGJ_2_, indicating a certain amount of 15d-PGJ_2_ inside of the erythrocytes is released into the plasma (ESM Table S1).

**Table 3:** Parameters of variability and extraction recovery for 15d-PGJ_2_

		Intraday			Interday		
Nominal concentration[pg mL^−1^]	Recovery [%]	Measured concentration [pg mL^−1^]	Accuracy [%]	Precision [% CV]	Measured concentration [pg mL^−1^]	Accuracy [%]	Precision [% CV]
5	91.6 ± 4.6	5.3 ± 0.6	106.0	11.3	4.9 ± 0.3	98	6.1
150	88.6 ± 13.9	141.2 ± 11.5	93.9	8.1	136.7 ± 15.8	91.1	11.6
400	96.1 ± 6.1	461.9 ± 54.3	115.5	11.8	389.1 ± 57.2	97.3	14.7

Displayed is the intra- and interday accuracy/precision and the extraction efficiency based on the recovery. Accuracy is defined as the mean of the quantified concentration in percent of 3 spiked concentrations (nominal) in human artificial plasma samples. Precision is described as the CV of the mean concentration determined for 3 different concentrations (intraday, *n* = 4; interday, n = 4)

### Stability

Pre-processing stability was evaluated by spiking human whole blood samples (500 μL) with 0.1 ng of 15d-PGJ_2_ and d4-15d-PGJ_2_. After storing for 1 or 6 h at ambient temperature (RT) or at 4 °C, samples were measured and resulting levels subtracted by the endogenous concentrations of 15d-PGJ_2_. Post-processing stability was documented after leaving samples for seven consecutive days in the autosampler at 4 °C or for 1 month at 4 °C. Stock and working solutions as well as pre-processed samples went through six freeze/thaw cycles to determine freeze/thaw stability (Table 4). The storage of working solutions in DMSO was associated with significantly lower instability for all compounds compared to other solvents (data not shown). In comparison to precursors of 15d-PGJ_2_, such as PGD_2_ or PGJ_2_ which undergo rapid degradation in aqueous solutions, we confirmed that 15d-PGJ_2_ is a stable metabolite in whole blood and assay buffer [22].

**Table 4:** Assessment of 15d-PGJ_2_ stabilities before extraction in whole blood (WB) or after extraction in assay buffer (AB) under varying conditions (ambient temperature (RT), 4 °C, − 20 °C; freeze/thaw (F/T))

Pre-processed (WB)				Post-processed (AB)				
1 h at RT	6 h at RT	1 h at 4 °C	6 h at 4 °C	1 week at 4 °C	1 month at 4 °C	1 week at − 20 °C	1 month at − 20 °C	F/T stability (6 cycles)
91%	89%	102%	95%	84%	76%	86%	91%	109%

Stability is defined as a change in percentage calculated by the measured concentrations divided by the nominal concentrations at *t* = 0 h (spiked with each analyte)

### Clinical application

**Fig. 3:**
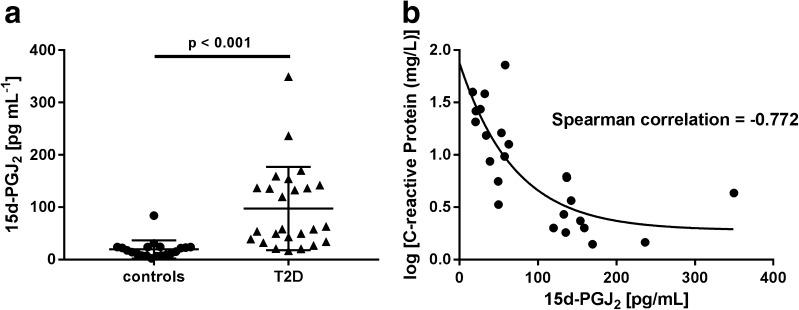
**A** 15d-PGJ_2_ plasma concentrations in controls and type 2 diabetic patients (T2D) with HbA_1c_ > 9%. **B** Correlation analysis of C-reactive protein (CRP) and 15d-PGJ_2_ in type 2 diabetic patients

This method was developed to allow the quantification of plasma 15d-PGJ_2_ in situations when the balance between pro- and anti-inflammatory properties is shifted toward inflammation. Therefore, the relation of CRP to 15d-PGJ_2_ was studied in healthy controls compared to patients suffering from type 2 diabetes. Baseline characteristics of the cohorts are summarized in Table 5. Utilizing the developed LC-MS/MS method, plasma levels of 15d-PGJ_2_ were found to be in the range of 2.5 to 349.6 pg mL^−1^, which are consistent with values previously reported in human plasma as measured by LC-MS/MS. For instance, PGD_2_, the precursor of 15d-PGJ_2_, has been quantified between 6 and 71 pg mL^−1^ [reviewed in 15]. Endogenous 15d-PGJ_2_ levels were mainly quantified in urine. Reported values for urinary 15d-PGJ_2_ levels were approximately 83 (GC-MS) and 6.3 (LC-MS) pg mg^−1^ creatinine [22, 23]. Unfortunately, as there are no studies which have determined 15d-PGJ_2_ in human plasma by LC-MS, the quantified concentrations in this study can only be compared to those made with enzyme immuno assays (EIA). In large cohorts (*n* = 200) of healthy controls and patients suffering from schizophrenia, 15d-PGJ_2_ was quantified in a range between 571 and 2577 pg mL^−1^ [24, 25]. These values are higher than the estimations given in this study and it is speculated that this could be the result of the very homogenous class of PGs resulting in a high degree of cross-reactivity with other PG species and therefore with unspecific EIA. In another study investigating plasma concentrations of 15d-PGJ_2_ in healthy volunteers and stroke patients, Blanco et al. reported levels between 3.8 and 109 pg mL^−1^ employing a different EIA [26]. This is in line with concentrations found in healthy controls and type 2 diabetic patients of our study. Furthermore, in the current study, 15d-PGJ_2_ was significantly elevated in type 2 diabetic patients and correlated negatively with the respective CRP value of each patient (Fig. 3a, b). 15d-PGJ_2_ has previously been shown to stimulate the anti-inflammatory transcription factor Nrf2 [5]. In diabetic animals, the overproduction of PGD_2_ (precursor of 15d-PGJ_2_) resulted in increased adipogenesis and improved insulin sensitivity, therefore counteracting the progression of diabetic complications [27–29]. Based upon these findings, our current study could point to an anti-inflammatory counterregulation mediated by elevated levels of the prostaglandin 15d-PGJ_2_. However, in a study of 15d-PGJ_2_ in human urine of obese and non-obese diabetic patients, there was no change, although it was not stated if the diabetic cohorts were in a high pro-inflammatory state [22]. Further investigations and validations with urine samples are necessary to determine the reasons for discrepancies in quantifying PGs under certain pathological conditions. Therefore, comparative measurements between GC-MS and LC-MS as well as urine and plasma samples need to be carried out in the near future.

**Table 5:** Mean baseline characteristics of the control and patient cohorts

Cohort (*n*)	Sex [% male]	Age [years]	Body weight [kg]	Height [cm]	BMI	Blood glucose [mg/dL]	HbA_1c_ [%]	CRP [mg/L]
Controls (20)	63.8	42.1 ± 12.3	71.3 ± 16.5	172.3 ± 12.9	24 ± 2.5	101.6 ± 26.6	4.5 ± 0.8	1.2 ± 1.1
Type 2 diabetic patients (25)	69.7	51.2 ± 13.4	101.4 ± 19.9	177.2 ± 12.1	32.4 ± 6.5**	186.4 ± 68.7	11.6 ± 2.1**	18.5 ± 10.1**

All parameters were determined prior to collection. Data are mean ± SD. Unless stated, all other characteristics were not significant (*p* > 0.05)

**p < 0.001, vs. controls

## Conclusion

This study describes a robust and sensitive LC-MS/MS method for the quantification of 15d-PGJ_2_ in human plasma. Detailed analytical parameters according to FDA guidance were determined and acceptable. Application of this method could contribute to an improved understanding of the physiological function of 15d-PGJ_2_. The preliminary findings in a small diabetic cohort may reflect an unrecognized counterregulation of systemic inflammation, potentially mediated by elevated levels of the anti-inflammatory prostaglandin 15d-PGJ_2_. Thus, the method presented here can be used in future studies to determine the balance of pro- and anti-inflammatory PGs under various physiological and pathological conditions.

## Electronic supplementary material

ESM 1 (PDF 437 kb)
