# Supplementary material for: Sensitive mass spectrometric assay for determination of 15-deoxy-Δ12,14-prostaglandin J2 and its application in human plasma samples of patients with diabetes
Source: Anal Bioanal Chem. 2017 Nov 16;410(2):521–8. doi: 10.1007/s00216-017-0748-1 (PMC5750338; doi:10.1007/s00216-017-0748-1)
Supplement: Supplementary file 1 — (PDF 437 kb) [file 216_2017_748_MOESM1_ESM.pdf]

Analytical and Bioanalytical Chemistry

Electronic Supplementary Material

Sensitive mass spectrometric assay for determination of 15-deoxy-$\Delta^{12,14}$-prostaglandin J_2 and its application in human plasma samples of patients with diabetes

Jakob Morgenstern, Thomas Fleming, Ivelina Kadiyska, Sebastian Brings,
Jan Benedikt Groener, Peter Nawroth, Markus Hecker, Maik Brune

Fig. S1

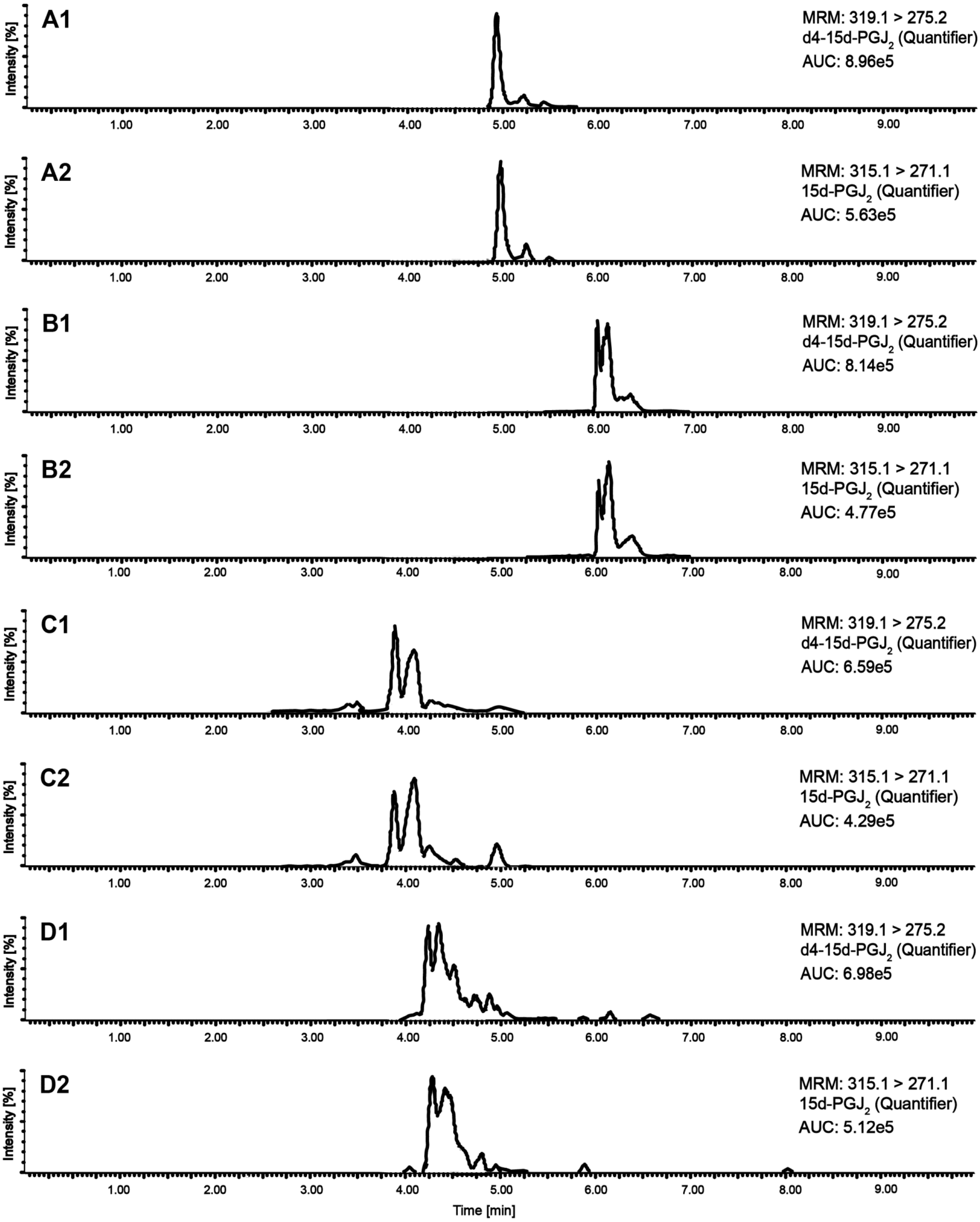

Electronic Supplementary Figure 1. Extracted MRM chromatograms (only quantifier) of 15d-PGJ$_2$ and its corresponding internal standard d4-15d-PGJ$_2$ of a calibration standard (50 pg mL^{-1} 15d-PGJ$_2$) under usage of different seperation columns.
A: Waters® Acquity BEH C18 column (1.7 µM, 2.1 x 100 mm).
B: Waters® Acquity BEH C18 column (1.7 µM, 2.1 x 150 mm).
C: Waters® Acquity BEH C18 column (2.5 µM, 2.1 x 50 mm).
D: Waters® Acquity BEH C18 column (1.7 µM, 2.1 x 50 mm)
+ Waters® Acquity BEH C18 VanGuard™ (5 x 2.1 mm; 1.7 µm)

Fig. S2

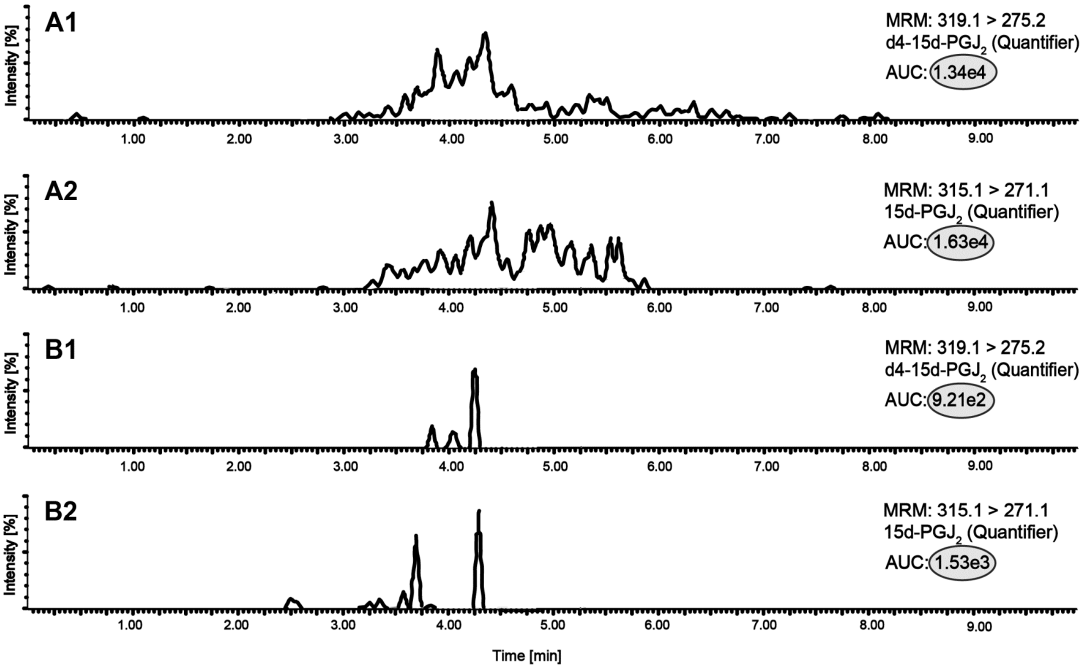

Electronic Supplementary Figure 2. Extracted MRM chromatograms (only quantifier) of 15d-PGJ_2 and its corresponding internal standard d4-15d-PGJ_2 of a blank injection without (A) or with (B) a 60 min isocratic washing run composed of H2O:ACN (1:1).

Table S1 Effect of hemolysis on quantification of 15d-PGJ_2. Displayed is the nominal (spiked) concentration in artificial plasma samples extracted and measured according to standard operating protocol (see 2.3). Samples were additionally spiked with increasing amounts of lysed erythrocytes and therefore hemoglobin. Percent recovery as determined by the division of nominal and measured concentrations of the internal standard. Values are given as the CV of the mean concentration ± SD (n = 3); **p* < 0.05

nominal concentration of 15d-PGJ_2 [pg mL^{-1}] + hemoglobin [mg dL^{-1}]	recovery [%]	measured concentration of 15d-PGJ_2 [pg mL^{-1}]
**150 + 10**	86.9 ± 2.8	145.1 ± 18.9
**150 + 50**	91.8 ± 10.2	161.8 ± 9.5
**150 + 500**	90.1 ± 7.3	189.3 ± 13.8 *
